# *N*,*N*,*N*′,*N*′-Tetra­benzyl-*N*′′-(2,6-difluoro­benzo­yl)phospho­ric triamide

**DOI:** 10.1107/S1600536812042481

**Published:** 2012-10-20

**Authors:** Akbar Raissi Shabari, Mehrdad Pourayoubi, Atekeh Tarahhomi, Arnold L. Rheingold, James A. Golen

**Affiliations:** a Faculty of Chemistry, North Tehran Branch, Islamic Azad University, Tehran, Iran; b Department of Chemistry, Ferdowsi University of Mashhad, Mashhad, Iran; c Department of Chemistry, University of California, San Diego, 9500 Gilman Drive, La Jolla, CA 92093, USA

## Abstract

In the C(O)NHP(O) fragment of the title compound, C_35_H_32_F_2_N_3_O_2_P, the P—N bond is longer and the O—P—N angle is contracted compared with the other two P—N bonds and O—P—N angles. The P atom adopts a distorted tetra­hedral environment and the phosphoryl and carbonyl groups are *anti* with respect to each other. The two tertiary N atoms of the dibenzyl­amido groups show *sp* ^2^ character with a slight deviation from planarity. In the crystal, pairs of N—H⋯O(P) hydrogen bonds form inversion dimers.

## Related literature

For related structures with a [C(O)NH]P(O)[N]_2_ configuration, see: Sabbaghi *et al.* (2010 ▶); Pourayoubi *et al.* (2010 ▶). For the geometries of the tertiary N atoms in phospho­ric triamides with a C(O)NHP(O)[N]_2_ core, see: Pourayoubi *et al.* (2012 ▶).
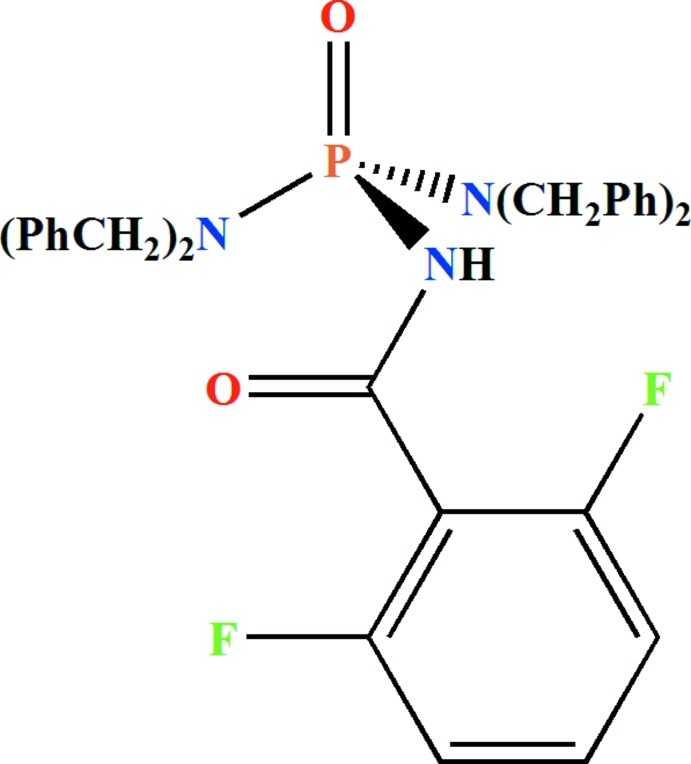


## Experimental

### 

#### Crystal data

C_35_H_32_F_2_N_3_O_2_P
*M* *_r_* = 595.61
Monoclinic, 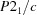

*a* = 12.3079 (7) Å
*b* = 19.5089 (12) Å
*c* = 13.0131 (6) Å
β = 105.430 (3)°
*V* = 3012.0 (3) Å^3^
*Z* = 4
Mo *K*α radiation
μ = 0.14 mm^−1^
*T* = 100 K
0.18 × 0.12 × 0.10 mm

#### Data collection

Bruker APEXII CCD diffractometer
Absorption correction: multi-scan (*SADABS*; Sheldrick, 2004 ▶) *T*_min_ = 0.975, *T*_max_ = 0.986
23928 measured reflections
6199 independent reflections
4332 reflections with *I* > 2σ(*I*)
*R*_int_ = 0.062

#### Refinement

*R*[*F* ^2^ > 2σ(*F* ^2^)] = 0.044
*wR*(*F* ^2^) = 0.111
*S* = 1.01
6199 reflections
391 parameters
1 restraint
H atoms treated by a mixture of independent and constrained refinement
Δρ_max_ = 0.28 e Å^−3^
Δρ_min_ = −0.39 e Å^−3^

### 

Data collection: *APEX2* (Bruker, 2005 ▶); cell refinement: *SAINT* (Bruker, 2005 ▶); data reduction: *SAINT*; program(s) used to solve structure: *SHELXS97* (Sheldrick, 2008 ▶); program(s) used to refine structure: *SHELXL97* (Sheldrick, 2008 ▶); molecular graphics: *SHELXTL* (Sheldrick, 2008 ▶); software used to prepare material for publication: *SHELXTL* and *enCIFer* (Allen *et al.*, 2004 ▶).

## Supplementary Material

Crystal structure: contains datablock(s) I, New_Global_Publ_Block. DOI: 10.1107/S1600536812042481/sj5269sup1.cif

Structure factors: contains datablock(s) I. DOI: 10.1107/S1600536812042481/sj5269Isup2.hkl

Additional supplementary materials:  crystallographic information; 3D view; checkCIF report

## Figures and Tables

**Table 1:** Hydrogen-bond geometry (Å, °)

*D*—H⋯*A*	*D*—H	H⋯*A*	*D*⋯*A*	*D*—H⋯*A*
N1—H1*N*⋯O2^i^	0.86 (2)	1.90 (2)	2.761 (2)	176 (2)

Symmetry code: (i) 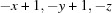.

**Fig. 1.**
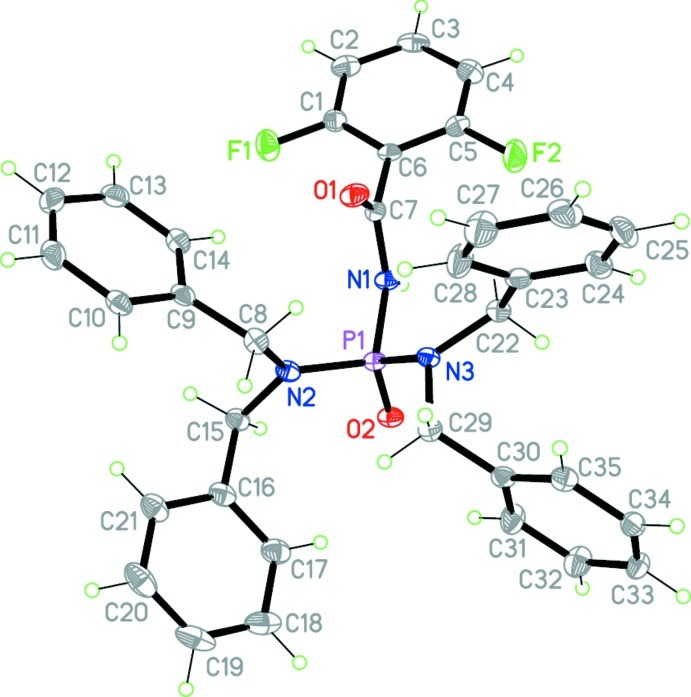
An ORTEP-style plot and atom labeling scheme for the title compound. Displacement ellipsoids are given at 50% probability level and H atoms are drawn as small spheres with arbitrary radii.

## supplementary crystallographic information

### Comment

The structure determination of the title compound, [2,6-F_2_—C_6_H_3_C(O)NH]P(O)[N(CH_2_C_6_H_5_)_2_]_2_ (Fig. 1), was performed as part of a project on the synthesis of new phosphoric triamides with a [C(O)NH]P(O)[N]_2_ skeleton, where '[N]_2_' denotes two tertiary N atoms belonging to an amide group.

The phosphoryl and carbonyl groups, which are separated by an N atom, have a relative *anti* disposition. In the C(O)NHP(O) fragment, the P1—N1 bond is longer and the O2—P1—N1 angle is contracted compared with the other two P—N bonds and O—P—N angles, similar to what is found for related phosphoramide derivatives (Sabbaghi *et al.*, 2010; Pourayoubi *et al.*, 2010).

The two tertiary N atoms show *sp*^2^ character with a slight deviation from planarity, wherein one of the two dibenzylamido N atoms shows a slightly greater deviation than the other [viz N3 with the sum of the surrounding bond angles = 353.48 (2)°]. The tertiary N3 atom is more pyramidal than N2 and is oriented so that the lone pair of electrons is *anti* with respect to the P═O group (Pourayoubi *et al.*, 2012).

In the crystal, the hydrogen atom of the C(O)NHP(O) moiety is involved in an intermolecular N—H···O(P) hydrogen bond (see Table 1) to form an inversion dimer.

### Experimental

2,6-F_2_—C_6_H_3_C(O)NHP(O)Cl_2_ was prepared according to a procedure reported by Pourayoubi *et al.* (2010).

To a solution of 2,6-F_2_—C_6_H_3_C(O)NHP(O)Cl_2_ (1.5 mmol) in chloroform (25 ml), a solution of dibenzylamine (6 mmol) in chloroform (5 ml) was added at 273 K. After 4 h stirring, the solvent was removed and the product was washed with distilled water and recrystallized from a mixture of CH_3_OH/CHCl_3_ (4:1 *v/v*) at room temperature.

### Refinement

The hydrogen atom H1N was found in a Fourier difference map and was allowed to refine isotropically with the N—H distance constrained to 0.87 (2) Å and *U*_iso_(H) = 1.2 *U*_eq_(N). All other hydrogen atoms were placed in calculated positions with appropriate riding parameters.

### Crystal data

**Table:**

C_35_H_32_F_2_N_3_O_2_P	*F*(000) = 1248
*M**_r_* = 595.61	*D*_x_ = 1.313 Mg m^−^^3^
Monoclinic, *P*2_1_/*c*	Mo *K*α radiation, λ = 0.71073 Å
Hall symbol: -P 2ybc	Cell parameters from 3822 reflections
*a* = 12.3079 (7) Å	θ = 2.7–25.0°
*b* = 19.5089 (12) Å	µ = 0.14 mm^−^^1^
*c* = 13.0131 (6) Å	*T* = 100 K
β = 105.430 (3)°	Block, colourless
*V* = 3012.0 (3) Å^3^	0.18 × 0.12 × 0.10 mm
*Z* = 4	

### Data collection

**Table:**

Bruker APEXII CCD diffractometer	6199 independent reflections
Radiation source: fine-focus sealed tube	4332 reflections with *I* > 2σ(*I*)
Graphite monochromator	*R*_int_ = 0.062
φ and ω scans	θ_max_ = 26.5°, θ_min_ = 2.9°
Absorption correction: multi-scan (*SADABS*; Sheldrick, 2004)	*h* = −15→15
*T*_min_ = 0.975, *T*_max_ = 0.986	*k* = −24→24
23928 measured reflections	*l* = −10→16

### Refinement

**Table:**

Refinement on *F*^2^	Primary atom site location: structure-invariant direct methods
Least-squares matrix: full	Secondary atom site location: difference Fourier map
*R*[*F*^2^ > 2σ(*F*^2^)] = 0.044	Hydrogen site location: inferred from neighbouring sites
*wR*(*F*^2^) = 0.111	H atoms treated by a mixture of independent and constrained refinement
*S* = 1.01	*w* = 1/[σ^2^(*F*_o_^2^) + (0.0493*P*)^2^ + 0.6985*P*] where *P* = (*F*_o_^2^ + 2*F*_c_^2^)/3
6199 reflections	(Δ/σ)_max_ = 0.001
391 parameters	Δρ_max_ = 0.28 e Å^−^^3^
1 restraint	Δρ_min_ = −0.39 e Å^−^^3^

### Special details

**Table:**

Experimental. IR (KBr, ν, cm^-1^): 3052, 2885, 1699, 1609, 1471, 1353, 1277, 1186, 1115, 1077, 1011, 930, 854, 802, 745.
Geometry. All e.s.d.'s (except the e.s.d. in the dihedral angle between two l.s. planes) are estimated using the full covariance matrix. The cell e.s.d.'s are taken into account individually in the estimation of e.s.d.'s in distances, angles and torsion angles; correlations between e.s.d.'s in cell parameters are only used when they are defined by crystal symmetry. An approximate (isotropic) treatment of cell e.s.d.'s is used for estimating e.s.d.'s involving l.s. planes.
Refinement. Refinement of *F*^2^ against ALL reflections. The weighted *R*-factor *wR* and goodness of fit *S* are based on *F*^2^, conventional *R*-factors *R* are based on *F*, with *F* set to zero for negative *F*^2^. The threshold expression of *F*^2^ > σ(*F*^2^) is used only for calculating *R*-factors(gt) *etc*. and is not relevant to the choice of reflections for refinement. *R*-factors based on *F*^2^ are statistically about twice as large as those based on *F*, and *R*- factors based on ALL data will be even larger.

### Fractional atomic coordinates and isotropic or equivalent isotropic displacement parameters (Å^2^)

**Table:**

	*x*	*y*	*z*	*U*_iso_*/*U*_eq_	
P1	0.59266 (4)	0.54027 (3)	0.16718 (4)	0.01441 (13)	
F1	0.85267 (11)	0.58682 (6)	−0.04221 (9)	0.0281 (3)	
F2	0.78767 (12)	0.36483 (7)	0.05916 (11)	0.0416 (4)	
O1	0.84569 (11)	0.52129 (7)	0.18588 (11)	0.0207 (3)	
O2	0.47271 (11)	0.53813 (7)	0.10611 (10)	0.0172 (3)	
N1	0.66497 (13)	0.50969 (8)	0.08388 (13)	0.0156 (4)	
H1N	0.6240 (16)	0.4957 (10)	0.0232 (13)	0.019*	
N2	0.62881 (13)	0.61912 (8)	0.20347 (12)	0.0155 (4)	
N3	0.63429 (13)	0.49405 (8)	0.27580 (12)	0.0149 (4)	
C1	0.85840 (17)	0.51846 (10)	−0.05653 (16)	0.0185 (4)	
C2	0.90366 (17)	0.49400 (11)	−0.13495 (16)	0.0226 (5)	
H2A	0.9310	0.5245	−0.1792	0.027*	
C3	0.90846 (17)	0.42409 (12)	−0.14784 (16)	0.0244 (5)	
H3A	0.9400	0.4062	−0.2014	0.029*	
C4	0.86834 (19)	0.37989 (12)	−0.08434 (18)	0.0288 (5)	
H4A	0.8703	0.3317	−0.0941	0.035*	
C5	0.82520 (18)	0.40748 (11)	−0.00619 (17)	0.0238 (5)	
C6	0.81866 (16)	0.47654 (10)	0.01110 (15)	0.0157 (4)	
C7	0.77876 (17)	0.50491 (10)	0.10290 (16)	0.0166 (4)	
C8	0.72603 (16)	0.63857 (10)	0.29096 (15)	0.0173 (4)	
H8A	0.7710	0.5970	0.3171	0.021*	
H8B	0.6984	0.6567	0.3504	0.021*	
C9	0.80209 (16)	0.69147 (10)	0.26126 (16)	0.0171 (4)	
C10	0.84585 (16)	0.74433 (10)	0.33157 (16)	0.0202 (5)	
H10A	0.8213	0.7496	0.3944	0.024*	
C11	0.92460 (18)	0.78945 (11)	0.31167 (18)	0.0253 (5)	
H11A	0.9546	0.8247	0.3614	0.030*	
C12	0.95970 (18)	0.78337 (11)	0.21971 (18)	0.0266 (5)	
H12A	1.0141	0.8141	0.2061	0.032*	
C13	0.91462 (17)	0.73200 (11)	0.14762 (17)	0.0238 (5)	
H13A	0.9376	0.7279	0.0837	0.029*	
C14	0.83644 (17)	0.68657 (10)	0.16778 (16)	0.0200 (5)	
H14A	0.8059	0.6517	0.1174	0.024*	
C15	0.55331 (17)	0.67630 (10)	0.15645 (15)	0.0173 (4)	
H15A	0.4978	0.6601	0.0912	0.021*	
H15B	0.5982	0.7133	0.1356	0.021*	
C16	0.49147 (17)	0.70473 (10)	0.23293 (15)	0.0187 (4)	
C17	0.39712 (17)	0.67077 (11)	0.24741 (16)	0.0233 (5)	
H17A	0.3712	0.6300	0.2086	0.028*	
C18	0.34077 (19)	0.69638 (12)	0.31862 (17)	0.0289 (5)	
H18A	0.2757	0.6735	0.3275	0.035*	
C19	0.3792 (2)	0.75492 (12)	0.37635 (17)	0.0314 (6)	
H19A	0.3412	0.7719	0.4258	0.038*	
C20	0.4724 (2)	0.78869 (12)	0.36247 (17)	0.0298 (6)	
H20A	0.4986	0.8290	0.4024	0.036*	
C21	0.52835 (19)	0.76405 (10)	0.29027 (16)	0.0227 (5)	
H21A	0.5920	0.7879	0.2802	0.027*	
C22	0.67520 (17)	0.42326 (10)	0.27705 (16)	0.0190 (4)	
H22A	0.7078	0.4168	0.2159	0.023*	
H22B	0.6104	0.3916	0.2675	0.023*	
C23	0.76288 (17)	0.40440 (10)	0.37836 (16)	0.0191 (5)	
C24	0.77030 (18)	0.33680 (11)	0.41232 (16)	0.0231 (5)	
H24A	0.7203	0.3035	0.3721	0.028*	
C25	0.84993 (19)	0.31741 (11)	0.50427 (17)	0.0263 (5)	
H25A	0.8552	0.2707	0.5258	0.032*	
C26	0.92163 (18)	0.36505 (12)	0.56484 (18)	0.0284 (5)	
H26A	0.9759	0.3514	0.6281	0.034*	
C27	0.91417 (19)	0.43264 (12)	0.53319 (19)	0.0330 (6)	
H27A	0.9623	0.4660	0.5754	0.040*	
C28	0.83611 (18)	0.45199 (11)	0.43947 (18)	0.0297 (5)	
H28A	0.8328	0.4984	0.4169	0.036*	
C29	0.57861 (17)	0.50956 (10)	0.36177 (15)	0.0169 (4)	
H29A	0.5423	0.5551	0.3480	0.020*	
H29B	0.6370	0.5121	0.4307	0.020*	
C30	0.49109 (16)	0.45744 (10)	0.37099 (15)	0.0162 (4)	
C31	0.38563 (18)	0.45573 (11)	0.29768 (17)	0.0235 (5)	
H31A	0.3682	0.4874	0.2402	0.028*	
C32	0.30626 (18)	0.40808 (11)	0.30833 (18)	0.0265 (5)	
H32A	0.2345	0.4073	0.2579	0.032*	
C33	0.33008 (18)	0.36155 (11)	0.39148 (16)	0.0215 (5)	
H33A	0.2748	0.3293	0.3987	0.026*	
C34	0.43417 (17)	0.36235 (10)	0.46344 (16)	0.0193 (4)	
H34A	0.4515	0.3303	0.5204	0.023*	
C35	0.51438 (17)	0.41011 (10)	0.45299 (15)	0.0184 (4)	
H35A	0.5864	0.4102	0.5030	0.022*	

### Atomic displacement parameters (Å^2^)

**Table:**

	*U*^11^	*U*^22^	*U*^33^	*U*^12^	*U*^13^	*U*^23^
P1	0.0122 (3)	0.0172 (3)	0.0140 (3)	0.0008 (2)	0.0036 (2)	−0.0007 (2)
F1	0.0412 (8)	0.0198 (6)	0.0268 (7)	0.0001 (6)	0.0154 (6)	0.0029 (5)
F2	0.0616 (10)	0.0199 (7)	0.0603 (9)	−0.0026 (6)	0.0459 (8)	0.0008 (6)
O1	0.0142 (7)	0.0271 (8)	0.0198 (8)	−0.0020 (6)	0.0027 (6)	−0.0038 (6)
O2	0.0133 (7)	0.0224 (7)	0.0157 (7)	0.0004 (6)	0.0034 (6)	−0.0037 (6)
N1	0.0120 (9)	0.0202 (9)	0.0142 (9)	0.0008 (7)	0.0028 (7)	−0.0015 (7)
N2	0.0139 (9)	0.0181 (9)	0.0135 (8)	0.0035 (7)	0.0018 (7)	0.0007 (7)
N3	0.0147 (9)	0.0157 (8)	0.0154 (9)	0.0019 (7)	0.0060 (7)	−0.0011 (7)
C1	0.0162 (11)	0.0189 (11)	0.0192 (11)	0.0005 (8)	0.0025 (9)	0.0006 (8)
C2	0.0170 (11)	0.0335 (13)	0.0169 (11)	0.0037 (9)	0.0039 (9)	0.0060 (9)
C3	0.0177 (11)	0.0370 (13)	0.0204 (11)	0.0052 (10)	0.0083 (9)	−0.0036 (10)
C4	0.0289 (13)	0.0242 (12)	0.0370 (13)	0.0029 (10)	0.0152 (11)	−0.0055 (10)
C5	0.0222 (12)	0.0226 (12)	0.0314 (13)	−0.0013 (9)	0.0157 (10)	0.0010 (9)
C6	0.0098 (10)	0.0202 (11)	0.0170 (10)	0.0014 (8)	0.0033 (8)	−0.0012 (8)
C7	0.0162 (10)	0.0148 (10)	0.0201 (11)	0.0005 (8)	0.0072 (9)	0.0030 (8)
C8	0.0189 (11)	0.0177 (10)	0.0131 (10)	0.0012 (8)	0.0002 (8)	−0.0017 (8)
C9	0.0134 (10)	0.0158 (10)	0.0202 (11)	0.0049 (8)	0.0011 (8)	0.0005 (8)
C10	0.0177 (11)	0.0188 (11)	0.0219 (11)	0.0050 (8)	0.0014 (9)	−0.0027 (8)
C11	0.0205 (12)	0.0184 (11)	0.0322 (13)	0.0002 (9)	−0.0014 (10)	−0.0043 (9)
C12	0.0164 (11)	0.0210 (11)	0.0418 (14)	−0.0001 (9)	0.0066 (10)	0.0042 (10)
C13	0.0200 (11)	0.0235 (12)	0.0290 (12)	0.0066 (9)	0.0086 (10)	0.0033 (9)
C14	0.0209 (11)	0.0172 (10)	0.0213 (11)	0.0026 (9)	0.0043 (9)	−0.0004 (8)
C15	0.0180 (11)	0.0176 (10)	0.0161 (10)	0.0042 (8)	0.0042 (8)	0.0027 (8)
C16	0.0194 (11)	0.0202 (11)	0.0153 (11)	0.0084 (8)	0.0026 (9)	0.0062 (8)
C17	0.0210 (12)	0.0259 (12)	0.0224 (12)	0.0069 (9)	0.0051 (9)	0.0043 (9)
C18	0.0230 (12)	0.0399 (14)	0.0265 (13)	0.0113 (10)	0.0115 (10)	0.0094 (10)
C19	0.0372 (14)	0.0391 (14)	0.0216 (12)	0.0224 (12)	0.0141 (11)	0.0070 (10)
C20	0.0453 (15)	0.0234 (12)	0.0193 (12)	0.0135 (11)	0.0063 (11)	0.0026 (9)
C21	0.0293 (12)	0.0200 (11)	0.0189 (11)	0.0078 (9)	0.0065 (9)	0.0046 (9)
C22	0.0185 (11)	0.0183 (10)	0.0220 (11)	0.0013 (8)	0.0085 (9)	0.0002 (9)
C23	0.0158 (11)	0.0216 (11)	0.0223 (11)	0.0031 (8)	0.0093 (9)	0.0028 (8)
C24	0.0275 (12)	0.0204 (11)	0.0235 (12)	0.0035 (9)	0.0104 (10)	−0.0012 (9)
C25	0.0343 (13)	0.0200 (11)	0.0268 (12)	0.0101 (10)	0.0118 (10)	0.0066 (9)
C26	0.0221 (12)	0.0338 (13)	0.0289 (13)	0.0105 (10)	0.0060 (10)	0.0104 (10)
C27	0.0229 (13)	0.0334 (13)	0.0374 (14)	−0.0028 (10)	−0.0009 (11)	0.0072 (11)
C28	0.0225 (12)	0.0242 (12)	0.0383 (14)	−0.0030 (9)	0.0009 (10)	0.0122 (10)
C29	0.0196 (11)	0.0174 (10)	0.0143 (10)	0.0002 (8)	0.0057 (8)	−0.0004 (8)
C30	0.0175 (11)	0.0174 (10)	0.0151 (10)	0.0003 (8)	0.0068 (8)	−0.0030 (8)
C31	0.0225 (12)	0.0221 (11)	0.0235 (11)	−0.0005 (9)	0.0016 (9)	0.0083 (9)
C32	0.0187 (11)	0.0280 (12)	0.0290 (12)	−0.0033 (9)	−0.0003 (10)	0.0050 (10)
C33	0.0222 (11)	0.0184 (11)	0.0261 (12)	−0.0045 (9)	0.0101 (9)	−0.0004 (9)
C34	0.0239 (12)	0.0180 (10)	0.0184 (11)	0.0030 (9)	0.0097 (9)	0.0035 (8)
C35	0.0196 (11)	0.0218 (11)	0.0140 (10)	0.0022 (8)	0.0051 (8)	−0.0003 (8)

### Geometric parameters (Å, º)

**Table:**

P1—O2	1.4794 (14)	C16—C21	1.386 (3)
P1—N2	1.6358 (17)	C16—C17	1.393 (3)
P1—N3	1.6395 (16)	C17—C18	1.390 (3)
P1—N1	1.6841 (17)	C17—H17A	0.9500
F1—C1	1.351 (2)	C18—C19	1.380 (3)
F2—C5	1.356 (2)	C18—H18A	0.9500
O1—C7	1.214 (2)	C19—C20	1.376 (3)
N1—C7	1.359 (2)	C19—H19A	0.9500
N1—H1N	0.860 (15)	C20—C21	1.390 (3)
N2—C8	1.465 (2)	C20—H20A	0.9500
N2—C15	1.476 (2)	C21—H21A	0.9500
N3—C22	1.469 (2)	C22—C23	1.510 (3)
N3—C29	1.490 (2)	C22—H22A	0.9900
C1—C2	1.372 (3)	C22—H22B	0.9900
C1—C6	1.383 (3)	C23—C24	1.386 (3)
C2—C3	1.377 (3)	C23—C28	1.387 (3)
C2—H2A	0.9500	C24—C25	1.382 (3)
C3—C4	1.374 (3)	C24—H24A	0.9500
C3—H3A	0.9500	C25—C26	1.376 (3)
C4—C5	1.376 (3)	C25—H25A	0.9500
C4—H4A	0.9500	C26—C27	1.377 (3)
C5—C6	1.372 (3)	C26—H26A	0.9500
C6—C7	1.512 (3)	C27—C28	1.389 (3)
C8—C9	1.511 (3)	C27—H27A	0.9500
C8—H8A	0.9900	C28—H28A	0.9500
C8—H8B	0.9900	C29—C30	1.509 (3)
C9—C10	1.389 (3)	C29—H29A	0.9900
C9—C14	1.393 (3)	C29—H29B	0.9900
C10—C11	1.384 (3)	C30—C35	1.382 (3)
C10—H10A	0.9500	C30—C31	1.392 (3)
C11—C12	1.381 (3)	C31—C32	1.382 (3)
C11—H11A	0.9500	C31—H31A	0.9500
C12—C13	1.384 (3)	C32—C33	1.383 (3)
C12—H12A	0.9500	C32—H32A	0.9500
C13—C14	1.384 (3)	C33—C34	1.371 (3)
C13—H13A	0.9500	C33—H33A	0.9500
C14—H14A	0.9500	C34—C35	1.390 (3)
C15—C16	1.510 (3)	C34—H34A	0.9500
C15—H15A	0.9900	C35—H35A	0.9500
C15—H15B	0.9900		
			
O2—P1—N2	109.73 (8)	C21—C16—C17	119.32 (19)
O2—P1—N3	119.07 (8)	C21—C16—C15	120.73 (19)
N2—P1—N3	105.64 (8)	C17—C16—C15	119.94 (18)
O2—P1—N1	105.49 (8)	C18—C17—C16	120.1 (2)
N2—P1—N1	111.66 (8)	C18—C17—H17A	120.0
N3—P1—N1	105.23 (8)	C16—C17—H17A	120.0
C7—N1—P1	127.05 (14)	C19—C18—C17	120.1 (2)
C7—N1—H1N	118.0 (14)	C19—C18—H18A	120.0
P1—N1—H1N	115.0 (14)	C17—C18—H18A	120.0
C8—N2—C15	114.85 (15)	C20—C19—C18	120.1 (2)
C8—N2—P1	124.82 (13)	C20—C19—H19A	119.9
C15—N2—P1	119.87 (13)	C18—C19—H19A	119.9
C22—N3—C29	114.04 (15)	C19—C20—C21	120.2 (2)
C22—N3—P1	123.66 (13)	C19—C20—H20A	119.9
C29—N3—P1	115.78 (12)	C21—C20—H20A	119.9
F1—C1—C2	119.50 (18)	C16—C21—C20	120.1 (2)
F1—C1—C6	117.12 (17)	C16—C21—H21A	119.9
C2—C1—C6	123.38 (19)	C20—C21—H21A	119.9
C1—C2—C3	118.3 (2)	N3—C22—C23	113.71 (16)
C1—C2—H2A	120.8	N3—C22—H22A	108.8
C3—C2—H2A	120.8	C23—C22—H22A	108.8
C4—C3—C2	121.0 (2)	N3—C22—H22B	108.8
C4—C3—H3A	119.5	C23—C22—H22B	108.8
C2—C3—H3A	119.5	H22A—C22—H22B	107.7
C3—C4—C5	118.0 (2)	C24—C23—C28	118.44 (19)
C3—C4—H4A	121.0	C24—C23—C22	118.76 (18)
C5—C4—H4A	121.0	C28—C23—C22	122.79 (18)
F2—C5—C6	117.08 (18)	C25—C24—C23	120.5 (2)
F2—C5—C4	119.10 (19)	C25—C24—H24A	119.8
C6—C5—C4	123.8 (2)	C23—C24—H24A	119.8
C5—C6—C1	115.49 (18)	C26—C25—C24	120.7 (2)
C5—C6—C7	122.33 (18)	C26—C25—H25A	119.7
C1—C6—C7	122.03 (18)	C24—C25—H25A	119.7
O1—C7—N1	124.51 (18)	C25—C26—C27	119.6 (2)
O1—C7—C6	120.86 (17)	C25—C26—H26A	120.2
N1—C7—C6	114.63 (17)	C27—C26—H26A	120.2
N2—C8—C9	114.30 (16)	C26—C27—C28	119.9 (2)
N2—C8—H8A	108.7	C26—C27—H27A	120.1
C9—C8—H8A	108.7	C28—C27—H27A	120.1
N2—C8—H8B	108.7	C23—C28—C27	120.9 (2)
C9—C8—H8B	108.7	C23—C28—H28A	119.5
H8A—C8—H8B	107.6	C27—C28—H28A	119.5
C10—C9—C14	118.14 (19)	N3—C29—C30	113.79 (15)
C10—C9—C8	119.78 (18)	N3—C29—H29A	108.8
C14—C9—C8	121.94 (17)	C30—C29—H29A	108.8
C11—C10—C9	121.1 (2)	N3—C29—H29B	108.8
C11—C10—H10A	119.4	C30—C29—H29B	108.8
C9—C10—H10A	119.4	H29A—C29—H29B	107.7
C12—C11—C10	120.2 (2)	C35—C30—C31	118.50 (19)
C12—C11—H11A	119.9	C35—C30—C29	120.37 (18)
C10—C11—H11A	119.9	C31—C30—C29	121.12 (18)
C11—C12—C13	119.2 (2)	C32—C31—C30	120.18 (19)
C11—C12—H12A	120.4	C32—C31—H31A	119.9
C13—C12—H12A	120.4	C30—C31—H31A	119.9
C14—C13—C12	120.6 (2)	C31—C32—C33	120.8 (2)
C14—C13—H13A	119.7	C31—C32—H32A	119.6
C12—C13—H13A	119.7	C33—C32—H32A	119.6
C13—C14—C9	120.63 (19)	C34—C33—C32	119.5 (2)
C13—C14—H14A	119.7	C34—C33—H33A	120.3
C9—C14—H14A	119.7	C32—C33—H33A	120.3
N2—C15—C16	112.12 (15)	C33—C34—C35	119.98 (19)
N2—C15—H15A	109.2	C33—C34—H34A	120.0
C16—C15—H15A	109.2	C35—C34—H34A	120.0
N2—C15—H15B	109.2	C30—C35—C34	121.09 (19)
C16—C15—H15B	109.2	C30—C35—H35A	119.5
H15A—C15—H15B	107.9	C34—C35—H35A	119.5
			
O2—P1—N1—C7	176.64 (16)	C10—C11—C12—C13	0.4 (3)
N2—P1—N1—C7	57.50 (19)	C11—C12—C13—C14	−0.9 (3)
N3—P1—N1—C7	−56.63 (18)	C12—C13—C14—C9	−0.3 (3)
O2—P1—N2—C8	159.51 (15)	C10—C9—C14—C13	2.0 (3)
N3—P1—N2—C8	29.97 (18)	C8—C9—C14—C13	−173.73 (19)
N1—P1—N2—C8	−83.90 (17)	C8—N2—C15—C16	−68.0 (2)
O2—P1—N2—C15	−12.28 (17)	P1—N2—C15—C16	104.57 (17)
N3—P1—N2—C15	−141.81 (14)	N2—C15—C16—C21	99.8 (2)
N1—P1—N2—C15	104.31 (15)	N2—C15—C16—C17	−79.4 (2)
O2—P1—N3—C22	90.02 (16)	C21—C16—C17—C18	0.1 (3)
N2—P1—N3—C22	−146.14 (15)	C15—C16—C17—C18	179.28 (18)
N1—P1—N3—C22	−27.88 (17)	C16—C17—C18—C19	−1.0 (3)
O2—P1—N3—C29	−59.88 (15)	C17—C18—C19—C20	1.0 (3)
N2—P1—N3—C29	63.95 (15)	C18—C19—C20—C21	0.0 (3)
N1—P1—N3—C29	−177.79 (13)	C17—C16—C21—C20	0.9 (3)
F1—C1—C2—C3	−179.99 (17)	C15—C16—C21—C20	−178.33 (18)
C6—C1—C2—C3	0.8 (3)	C19—C20—C21—C16	−0.9 (3)
C1—C2—C3—C4	0.4 (3)	C29—N3—C22—C23	−61.0 (2)
C2—C3—C4—C5	−1.2 (3)	P1—N3—C22—C23	148.61 (14)
C3—C4—C5—F2	−178.4 (2)	N3—C22—C23—C24	150.43 (18)
C3—C4—C5—C6	0.8 (3)	N3—C22—C23—C28	−29.2 (3)
F2—C5—C6—C1	179.56 (18)	C28—C23—C24—C25	−0.8 (3)
C4—C5—C6—C1	0.3 (3)	C22—C23—C24—C25	179.58 (18)
F2—C5—C6—C7	3.9 (3)	C23—C24—C25—C26	1.3 (3)
C4—C5—C6—C7	−175.3 (2)	C24—C25—C26—C27	−0.3 (3)
F1—C1—C6—C5	179.63 (17)	C25—C26—C27—C28	−1.3 (4)
C2—C1—C6—C5	−1.2 (3)	C24—C23—C28—C27	−0.8 (3)
F1—C1—C6—C7	−4.7 (3)	C22—C23—C28—C27	178.8 (2)
C2—C1—C6—C7	174.50 (18)	C26—C27—C28—C23	1.8 (4)
P1—N1—C7—O1	1.6 (3)	C22—N3—C29—C30	−48.9 (2)
P1—N1—C7—C6	−178.72 (14)	P1—N3—C29—C30	103.88 (17)
C5—C6—C7—O1	95.9 (2)	N3—C29—C30—C35	104.5 (2)
C1—C6—C7—O1	−79.5 (3)	N3—C29—C30—C31	−75.5 (2)
C5—C6—C7—N1	−83.8 (2)	C35—C30—C31—C32	0.8 (3)
C1—C6—C7—N1	100.8 (2)	C29—C30—C31—C32	−179.23 (19)
C15—N2—C8—C9	−58.6 (2)	C30—C31—C32—C33	0.0 (3)
P1—N2—C8—C9	129.21 (16)	C31—C32—C33—C34	−0.7 (3)
N2—C8—C9—C10	138.58 (18)	C32—C33—C34—C35	0.6 (3)
N2—C8—C9—C14	−45.8 (2)	C31—C30—C35—C34	−0.9 (3)
C14—C9—C10—C11	−2.4 (3)	C29—C30—C35—C34	179.13 (18)
C8—C9—C10—C11	173.37 (18)	C33—C34—C35—C30	0.2 (3)
C9—C10—C11—C12	1.2 (3)		

### Hydrogen-bond geometry (Å, º)

**Table:**

*D*—H···*A*	*D*—H	H···*A*	*D*···*A*	*D*—H···*A*
N1—H1*N*···O2^i^	0.86 (2)	1.90 (2)	2.761 (2)	176 (2)

Symmetry code: (i) −*x*+1, −*y*+1, −*z*.
